# Supporting families to achieve a healthy weight development for their child with overweight/obesity using the STARKIDS intervention: study protocol for a cluster-randomized controlled trial

**DOI:** 10.1186/s13063-022-06525-0

**Published:** 2022-07-23

**Authors:** Katrin Ziser, Florian Junne, Anne Herschbach, Peter Martus, Johann Jacoby, Felicitas Stuber, Zahra Rahmani Azad, Isabelle Mack, Alisa Weiland, Inga Krauß, Constanze Greule, Gorden Sudeck, Lydia Kastner, Guido Zurstiege, Andreas Hoell, Wolfgang Bethge, Torben Sammet, Olaf Schliesing, Stephan Zipfel, Stefan Ehehalt, Katrin E. Giel

**Affiliations:** 1 Department of Psychosomatic Medicine and Psychotherapy, Medical University Hospital Tuebingen, Tuebingen, Germany; 2 Centre of Excellence for Eating Disorders Tuebingen (KOMET), Tuebingen, Germany; 3 Department of Psychosomatic Medicine and Psychotherapy, Otto von Guericke University Magdeburg, Magdeburg, Germany; 4 Institute of Clinical Epidemiology and Applied Biometry, Medical Faculty, University of Tuebingen, Tuebingen, Germany; 5 Department of Sports Medicine, Medical University Hospital Tuebingen, Tuebingen, Germany; 6 Institute of Sports Science, University of Tuebingen, Tuebingen, Germany; 7 Insitute of Media Studies, University of Tuebingen, Tuebingen, Germany; 8 Clinic for Psychiatry and Psychotherapy, Central Institute of Mental Health, Medical Faculty Mannheim, University of Heidelberg, Mannheim, Germany; 9 Center for Clinical Trials, Medical Faculty, University of Tuebingen, Tuebingen, Germany; 10 Ministry of Social Affairs, Health and Integration Baden-Wuerttemberg, Stuttgart, Germany; 11 CES Computer Educations Systems GmbH, Heidelberg, Germany; 12 Public Health Department of Stuttgart, Stuttgart, Germany

**Keywords:** Childhood, Adolescent, Overweight, Obesity, e-Health, Pediatricians, Family-centered, Quality of life, Body weight, Diet, Physical activity, Media consumption, Lifestyle intervention

## Abstract

**Background:**

Childhood and adolescent overweight and obesity are among the most serious health challenges today. Structured weight reduction programs can be helpful to reduce severe health consequences but evidence is partly scarce. The STARKIDS program aims to improve on some of these limitations and is designed to be a structured, stepwise, digitally supported intervention program for the whole family. It is divided into two intervention steps spanning over 1.5 years and aims at promoting a healthy weight development of children/adolescents with overweight/obesity and an increase in quality of life.

**Methods:**

The STARKIDS intervention is evaluated in a cluster-randomized study design by comparing it with a control group receiving a one-time structured counselling in the pediatric practice. The study aims to include 1000 families with children/adolescents with overweight/obesity from 100 pediatric practices. The main outcomes are reduction in body mass index percentiles and improvements in children’s/adolescent’s quality of life, secondary outcomes refer to the contents of the intervention such as diet, physical activity, stress, and media habits. All outcomes are measured on three measurement time points: (T0) at baseline/inclusion in the study, (T1) baseline + 12 months which is the end of step 1 of the STARKIDS intervention, and (T2) baseline + 18 months which is the end of step 2 of the STARKIDS intervention.

**Discussion:**

The stepwise, e-health-supported STARKIDS program is a low-threshold intervention program for families with children/adolescents with overweight/obesity. With the proof of concept, STARKIDS provides the potential to be implemented as a standard care tool for the prevention and intervention of childhood/adolescence obesity in the German health system.

**Trial registration:**

German Clinical Trials Register (DRKS) DRKS00022813 (acknowledged primary register of the World Health Organization). Registered on 27 November 2020 (Universal Trial Number U1111-1254-9536).

## Administrative information

Note: The numbers in curly brackets in this protocol refer to SPIRIT checklist item numbers. The order of the items has been modified to group similar items (see http://www.equator-network.org/reporting-guidelines/spirit-2727-statement-defining-standard-protocol-items-for-clinical-trials/).

**Table:** 

Title {1}	Supporting families to achieve a healthy weight development for their child with overweight/obesity using the STARKIDS intervention: study protocol for a cluster-randomized controlled trial
Trial registration {2a and 2b}	This trial is registered with the German Clinical Trials Register (DRKS) under the trial number DRKS00022813 (registration date: 27.11.2020; Universal Trial Number U1111-1254-9536).
Protocol version {3}	November 12, 2021. Version 1.
Funding {4}	The current project is funded by the Innovation Committee of the German Joint Federal Committee (G-BA) with the funding number 01NVF18013 (STARKIDS).
Author details {5a}	^1^Department of Psychosomatic Medicine and Psychotherapy, Medical University Hospital Tuebingen, Tuebingen, Germany ^2^Centre of Excellence for Eating Disorders Tuebingen (KOMET), Tuebingen, Germany ^3^Department of Psychosomatic Medicine and Psychotherapy, Otto von Guericke University Magdeburg, Magdeburg, Germany ^4^Institute of Clinical Epidemiology and Applied Biometry, Medical Faculty, University of Tuebingen, Tuebingen, Germany ^5^Department of Sports Medicine, Medical University Hospital Tuebingen, Tuebingen, Germany ^6^Institute of Sports Science, University of Tuebingen, Tuebingen, Germany ^7^Insitute of Media Studies, University of Tuebingen, Tuebingen, Germany ^8^Clinic for Psychiatry and Psychotherapy, Central Institute of Mental Health, Medical Faculty Mannheim, University of Heidelberg, Mannheim, Germany ^9^Center for Clinical Trials, Medical Faculty, University of Tuebingen, Tuebingen, Germany ^10^Baden-Wuerttemberg State Health Office, Stuttgart, Germany ^11^CES Computer Educations Systems GmbH, Heidelberg, Germany ^12^Public Health Department of Stuttgart, Stuttgart, Germany
Name and contact information for the trial sponsor {5b}	Medical University Hospital Tuebingen Department of Psychosomatic Medicine and Psychotherapy Prof. Dr. med. Florian Junne Osianderstr. 5 72076 Tuebingen Germany florian.junne@med.ovgu.de
Role of sponsor {5c}	Role of study sponsor: role and ultimate authority in study design; collection, management, analysis, and interpretation of the data; writing of the report; and the decision to submit the report for publication. Role of study funder: no roles in the collection, management, analysis, and interpretation of the data; writing of the report; or the decision to submit the report for publication.

## Introduction

### Background and rationale {6a}

Overweight and obesity are among the major health concerns of today’s society. According to the World Health Organization (WHO), obesity has nearly tripled since 1975 [1]. Thirty-nine percent of adults worldwide are considered overweight [1] and thirteen percent obese [1, 2]. Overweight in adults is commonly measured through the body mass index (BMI) which is calculated as a division of a person’s weight through the squared height (kg/m^2^). Adults with a BMI equal to or greater than 25 kg/m^2^ are considered overweight, and adults with a BMI equal to or greater than 30 kg/m^2^ are considered having obesity. For children, overweight and obesity are diagnosed by comparison with norm data, taking children’s age and gender into account. This results in BMI percentiles. According to the German guidelines for treatment and prevention of obesity in childhood and adolescence, suitable reference values for German children/adolescents are a BMI percentile above 90 for overweight and a BMI percentile above 97 for obesity [3]. In 2016, 18% of girls and 19% of boys worldwide were overweight [1] and about 6% of girls about 8% of boys were obese [2, 4].

The rates of overweight/obesity in childhood and adolescence are a cause for concern because overweight/obesity often continues on into adulthood. In a review, Simmonds et al. [5] found a strong association between childhood obesity and adulthood obesity: children with obesity were five times more likely to become adults with obesity than children with no obesity. In addition, children with overweight and obesity have an increased risk for sequelae. These can be somatic, such as cardiovascular diseases, asthma, musculoskeletal pain, or even premature death, as well as psychological, such as low self-esteem and low quality of life [6–10]. From a health economic perspective, there are furthermore significant (lifetime) indirect costs of obesity [10, 11]. In light of these negative consequences, effective treatments for overweight and obesity in children and adolescents are urgently needed.

Over the last years, a great amount of weight reduction programs for children and adolescents have been developed, evaluated, and summarized in systematic reviews. Rajjo et al. [12] for example compared different therapeutic methods to reduce children’s weight and found that nonsurgical, multicomponent interventions combining diet, physical activity, and behavioral therapy were most effective. The first Cochrane review about the effectiveness of childhood weight reduction programs similarly concluded that combined behavior programs can lead to a reduction of body weight [13]. Ells et al. [14] updated this review in an overview of six Cochrane reviews and found similar effects: Multicomponent interventions led to reductions in body weight of children and adolescents with overweight or obesity of all age groups. The best supported interventions for weight loss in children and adolescents with overweight and obesity seem therefore multicomponent interventions addressing nutrition, physical activity as well as behavior.

Despite the support for multicomponent interventions, there are still substantial shortcomings. Ells et al. [14] conclude that the observed body weight reductions are small. Kobes et al. [15] conducted a quantitative meta-synthesis of 26 meta-analyses and found a standardized mean difference of − 0.12 BMI change between intervention and control groups. This indicates only a small change and the authors themselves question its clinical relevance. Other Cochrane reviews [16, 17] limit their conclusions towards the effectiveness of investigated interventions due to lacking trial quality and inconsistency of results. Mead et al. [18] refer to another shortcoming of previous weight reduction programs focusing on BMI (percentiles) only. They call for interventions targeting on additional variables such as quality of life and comorbidities. In summary, although there is a great amount of weight reduction programs for children and adolescents, most evaluating studies show insufficient quality and rather small effects. However, new approaches combining face-to-face counseling in low-barrier environments such as the pediatric practice in combination with personalized e-health interventions such as the STARKIDS program presented in the current protocol are scarce in the treatment of childhood overweight/obesity.

The aim of the STARKIDS program is to support families in achieving a healthy weight management and lifestyle for their children and adolescents with overweight/obesity. STARKIDS addresses children and adolescents with overweight/obesity between 3 and 17 years in the context of their whole family. The intervention lasts for one and a half years and is divided into two steps: the first step lasts 1 year and combines face-to-face interactions conducted in the pediatric practice with an e-health online platform to be used between the visits. If weight development is insufficient in step one, participating families receive further personal counselling by the public health department for another 6 months. A more detailed description of the STARKIDS program can be found in the course of this study protocol.

### Objectives {7}

The STARKIDS study, described in the present study protocol, aims to include 1000 children and adolescents and their families into a cluster-randomized controlled trial. It thereby compares weight development and quality of life as well as other variables associated with a healthy weight development between an intervention group receiving the newly developed combined approach of structured face-to-face and e-health-supported STARKIDS intervention and a control group receiving structured standard care.

The main hypotheses of the STARKIDS study are that the intervention group shows better outcomes in weight development and positive change in the quality of life in comparison with control. Further hypotheses are related to the superiority of the intervention for measures capturing healthy lifestyle habits.

### Trial design {8}

The STARKIDS study is a cluster-randomized controlled trial with two parallel study arms: The intervention group receives the new STARKIDS program whereas the control group receives treatment-as-usual (TAU). Both treatments are conducted in pediatric practices that are randomly allocated to either the intervention or the control condition. Participants are therefore cluster-randomized as their allocation depends on their practice being an intervention or a control practice.

Data is assessed at three measurement time points from participating pediatric practices as well as participating families:

- T0: baseline/inclusion in the study
- T1: T0 + 12 months, end of step 1 of the STARKIDS program
- T2: T0 + 18 months, end of step 2 of the STARKIDS program

The STARKIDS intervention is hypothesized to show superiority over TAU at T1 and T2.

## Methods: participants, interventions, and outcomes

### Study setting {9}

Data will be collected in the federal state of Baden-Wuerttemberg in the southwest of Germany through participating pediatric practices. Additionally, data will be collected from participating families through the STARKIDS online platform.

### Eligibility criteria {10}

Eligible for the study are families with children/adolescents with overweight/obesity. Since STARKIDS is a family-centered intervention, at least one parent/legal guardian and the child/adolescent with overweight/obesity are included in the study. If a family has more than one eligible child/adolescent, one child/adolescent among the siblings is randomly chosen and included in the study. However, the whole family is able to participate in face-to-face counseling sessions.

#### Inclusion criteria

Families must meet the following inclusion criteria to be eligible to participate in the study:

- At least one child or adolescent between 3 and 17 years with overweight (body mass index > 90th percentile) or obesity (body mass index > 97th percentile) (cutoffs according to the German guidelines for treatment and prevention of obesity in childhood and adolescence [3])
- In the statutory health insurance and enrolled in a special program called “family doctor program” of the health insurance company AOK Baden-Wuerttemberg (pediatric module)
- Adequate knowledge of the German language (need the ability to understand the face-to-face counselling sessions and the e-health materials in German)

#### Exclusion criteria

If participants meet any of the following exclusion criteria, they cannot be enrolled in the study:

- Prior participation in an interdisciplinary obesity training for the duration of 1 year or more
- Participation in a child rehabilitation or inpatient treatment for weight loss within the past year
- Severe other systemic diseases of the child/adolescent (e.g., metabolic diseases including diabetes mellitus, oncological diseases, severe cardiovascular or respiratory diseases, and disorders of the nervous system or the musculoskeletal system)
- Severe other psycho-social stress or disease of the child/adolescent which is not associated with overweight/obesity (e.g., treatment of a schizophrenia spectrum disorder with appetite-enhancing medication such as neuroleptic drugs)

#### Eligibility criterion of the participating pediatric practices who will perform the interventions

- Enrollment in the pediatric module of the “family doctor program” of the health insurance company AOK Baden-Wuerttemberg (for an explanation of the family doctor program, see above)

### Who will take informed consent? {26a}

Informed consent of participating families will be obtained by participating pediatricians after informing about the study and answering families remaining questions. Information about the study and consent are given to parents as well as children and adolescents, age-appropriate information materials are provided for the following groups: parents/legal guardians, 6–11 years old children, and 12–17 years old adolescents. For families with a child under 6, only parents give informed consent. Pediatricians’ consent to participate in the study will be obtained by the study team.

### Additional consent provisions for collection and use of participant data and biological specimens {26b}

In addition to informed consent, pediatricians also obtain consent for the handling of families’ study data as well as retrieval of pre-defined medical data from the families’ health insurance records.

### Interventions

#### Explanation for the choice of comparators {6b}

In the absence of standardized care for children and adolescents with overweight and obesity in the German health system, the treatment-as-usual comparator group was chosen as a basic intervention giving recommendations about improving factors known to be associated with overweight/obesity in a one-time structured counseling.

#### Intervention description {11a}

Families in the control group receive a face-to-face one-time structured counseling (approximately 30 min) concerning topics shown to have an essential impact on healthy weight development. Similar to the STARKIDS program, the control counselling is provided by medical assistants in participating pediatric practices and addresses the topics healthy weight development, diet, physical activity, media consumption, sleeping habits, and stress. In contrast to the STARKIDS program, the control counselling is limited to providing guideline-based recommendations for each of the covered topics and does not provide further tools such as educative texts, serious games and reflection tools. Families in the control condition receive a flyer summarizing the guideline-based recommendations for a healthy weight development at the end of their one-time structured counseling.

Families in the intervention group receive the newly developed STARKIDS program. The STARKIDS program consists of two steps. The first step consists of face-to-face trainings in the pediatric practice and an e-health online platform. Both include the following modules: “STARKIDS Start,” “eating and drinking,” “activity and media,” “family life,” “STARKIDS Keep on,” and the optional “STARKIDS Joker.” For more details on the individual modules, see Table 1. Every 3 months, families undergo a 90-min-long face-to-face training each covering one module (in the given order) in their pediatric practice, thus continuing for 1 year from first to last face-to-face training. All trainings are taught by a medical assistant and are conducted in the spirit of motivational interviewing [19]. Motivational interviewing is a person-centered counseling style aimed at reducing ambivalence and strengthening internal motivation and commitment to change. The “STARKIDS Joker” is an optional sixth face-to-face training that can be activated by the medical assistant or participating families in case of motivational problems or other issues a family may encounter when implementing the program into their everyday life. This training can be held at any time in between the first and the last training (STARKIDS Start and STARKIDS Keep on).

**Table 1:** Overview of the thematic modules in the STARKIDS intervention

Overview	Concept(s)	Educative/interactive texts	Animated films	Reflection tools	Serious games
**STARKIDS Start**					
Introduction to the program	/	X			
Information about weight development in childhood and adolescence	/	X	X		
Introduction eating and drinking	- Meal frequency and timing - Drinks, with emphasis on sweet beverages - Composition of a healthy diet - Frequently asked questions about drinks and foods	X	X	X	X
Introduction activity and media	- Benefits of physical activity - Adequate amounts of physical activity - Opportunities for physical activity and opportunities to discover new places for physical activity - Dealing with barriers and obstacles for physical activity - Adequate times of media consumption - Identifying marketing mechanisms for unhealthy foods and drinks - Tips for media consumption	X	X	X	X
Introduction family life	- Family as a support system - Listening to each other - Joint family activities - Eating meals together - Consolation and reward (other than food)	X	X	X	
Introduction in mindfulness	- Basic information about mindfulness and how it can support a health weigh development process	X			X
**Eating and drinking**					
Information about the dietary concept in STARKIDS	- Why strict diets do not work - What is different in STARKIDS	X	X		
Meal frequency and timing	- Information on meal frequency and timing - Frequently asked questions on this topic	X	X	X	
Drinks	- Drinks, with emphasis on sweet beverages - Frequently asked questions on the topic	X	X	X	X
Primary focus: energy density	- Explanation of the dietary energy density concept - Practical use of the dietary energy concept in daily life - How to make clever food choices based on the dietary energy density concept - Frequently asked questions on the topic	X	X	X	X
Portion sizes	- Information about adequate portion sizes - Examples and tips for choosing adequate meals and snacks	X			
Tips for everyday life	- Tips for grocery shopping - Frequently asked questions about everyday meals - Information about the Nutri-Score	X	X		
Uncontrolled eating	- Information about binge eating, emotional eating, night eating, and grazing - Strategies for avoiding uncontrolled eating - Information for further help in case of eating disorder pathology	X			
Mindful eating and drinking	- Information about how mindfulness can support healthy saturation and indulgence	X			X
**Activity and media**					
Enjoying physical activity	- Information about fitness, energy consumption, and bodily reactions to physical activity - Supporting the fit between individual motives and corresponding possibilities to be physically active - Overview of different types of sports and exercise - Offering autonomous activity choices	X	X	X	X
Exploring locations for physical activity	- Tips for different locations for physical activity - Information about cycling - Strategies for integrating more physical activity in everyday life - Reflecting individual locations for physical activity	X	X	X	
Being physically active together	- Considering possible buddies for being active together, e.g., in clubs, with family members, in the neighborhood - Promotion of family activities - Illustrating role models from the obesity context to enhance the feeling of relatedness	X	X	X	X
Becoming physically active (in daily life, in leisure time, in sports clubs or schools)	- Information about age-appropriate amounts of physical activity - Tips for keeping motivated for physical activity in everyday life - Summary and tips for parents to support their children/adolescents - Reflection of and individual feedback about the amount of weekly physical activities	X	X	X	X
Media consumption	- Information about the age-appropriate amounts of screen time - Tips for children’s media consumption - Identifying marketing mechanisms for unhealthy foods on TV, social media, grocery shopping - Introduction to compulsive gambling and further help	X	X		X
Mindful activity and media consumption	- Information about how mindfulness can help through a balanced amount of activity and rest	X			X
**Family life**					
Strengthening family life	- Listening to each other - Talking about difficult topics - Strengthening family cohesion	X	X	X	
Dealing with stress	- Associations between stress and overweight/obesity - Identifying emotions - Possibilities for dealing with difficult emotions, negative thoughts and stress	X	X	X	
Sleeping habits	- Associations between stress, sleep, and weight increase - Tips for helpful sleeping habits	X	X		X
Weight stigmatization	- Individual appearances - Tips for dealing with stigmatization	X			
Self-worth	- Developing a positive relationship to oneself - Strengthening a positive body image	X			X
Mindful dealing with oneself	- Information how mindfulness can support personal changes and healthy sleep	X			X
**STARKIDS Keep on**					
Reflection of the last year with the STARKIDS program	/	X			
Dealing with setbacks	- Identifying risk situations and setbacks - Reflections of strategies against setbacks - Strategies for sustained change	X			
**STARKIDS Joker**					
Strengthening motivation and commitment	- Reflecting individual obstacles and reasons for staying with the program - Developing strategies to strengthen commitment - Tips for introducing change one step at a time	X		X	X

Face-to-face trainings follow a pre-defined sequence containing:

- Tablet-based pre-assessment: families indicate their previous behavior on the topic of the module, identify problem areas in this topic and rate their current motivational stage
- Discussing progress concerning goals and weight trend, problems, and obstacles since the last face-to-face training
- Educational input by the medical assistant about the topics of the current module under consideration of the results of families’ pre-assessments
- Choosing three individual module-specific goals and a weight goal for the next 3 months

The e-health online platform covers different resources allocated to the six modules. Families can watch educative movies, read and interact with educative texts, play serious games, use reflection tools, and utilize so-called tips paper as helpful reminders for changes in everyday life. They are asked to engage with content and different tasks of the respective module and are biweekly reminded to monitor their current goals and the current weight of the child/adolescent.

Step 1 of the STARKIDS program lasts 1 year. To evaluate step 1 of the STARKIDS program and to decide if participants should make use of step 2, pre-defined criteria of BMI reduction is applied_._ Children and adolescents that did not reduce their BMI by the predefined amount receive counselling about further programs and opportunities nearby, depending on the area families are still struggling with. The counselling is given by participating public health services in step 2 (1–2 counselling sessions in a 6-month time frame). Additionally, all families can carry on using the STARKIDS online platform for the next six months after which the program is concluded.

#### Criteria for discontinuing or modifying allocated interventions {11b}

In case participating families experience difficulties during step 1 of the STARKIDS intervention, the optional face-to-face training “STARKIDS Joker” can be activated by the family or the medical assistant in which difficulties are discussed and counteractive measures are identified and implemented. The criteria for discontinuing the intervention are families in need for more intensive treatment settings, e.g., due to occurring mental illnesses. The decision about the utilization of additional forms of treatment rests upon the attending pediatrician.

#### Strategies to improve adherence to interventions {11c}

To enhance adherence to intervention protocols, monetary compensation is provided to families for conducting questionnaires on each of the three measurement time points. Additionally, face-to-face trainings are conducted in the spirit of motivational interviewing thought to enhance compliance and coherence [19]. Furthermore, the STARKIDS online platform provides the possibility to send automated text messages reminding the family to use the platform, report their weight or reflect on their goals. As a further measure to improve adherence, participating pediatric practices can monitor their participating families’ use of the e-health online platform (last login and if questionnaires were completed) and are therefore able to actively approach inactive families.

#### Relevant concomitant care permitted or prohibited during the trial {11d}

In accordance with the exclusion criteria for the study, participation in an interdisciplinary obesity training for the duration of 1 year or more other than the STARKIDS program and participation in a child rehabilitation or inpatient treatment for weight loss are prohibited during the trial. Beyond these exclusions, implementing the structured face-to-face and e-health supported STARKIDS intervention or structured standard care will not require alteration to all other usual care pathways (including use of any medication) and these will continue for both trial arms.

#### Provisions for post-trial care {30}

Not applicable, since ancillary and post-trial care is provided within the standard care.

### Outcomes {12}

All outcomes are measured on three measurement time points: (T0) at baseline/inclusion in the study, (T1) baseline + 12 months, and (T2) baseline + 18 months. T1 marks the primary outcome measurement time point and T2 marks the secondary outcome measurement time point.

#### Primary outcomes

There are two hierarchically structured primary outcomes: an objective parameter measuring weight development as the main primary outcome and a subjective parameter measuring self- and parent-reported quality of life of the children and adolescents. Weight development is measured from baseline to follow-up in BMI standard deviation scores (BMI-SDS or BMI *z*-scores) that are derived using the LMS method [20] (BMI-SDS_LMS_) with a representative German reference population [21, 22]. The (hierarchically structured) second primary outcome is a positive change of mean in children’s and adolescents’ quality of life from baseline to follow-up [23]. Primary outcomes are assessed at each of the three measurement points in the pediatric practice (BMI-SDS_LMS_) or online via a questionnaire (quality of life).

#### Secondary outcomes

Secondary outcomes originate from a questionnaire which are answered at the three main measurement points and most commonly analyzed as change from baseline. Most variables are assessed twice: once in the parent perspective and once as an own perspective by children aged twelve and older. The secondary outcomes are structured around the main modules of the STARKIDS program. Table 2 contains a detailed overview of outcomes according to intervention modules including their psychometric properties. In summary, the following topics are covered:

- “Eating and drinking”: dietary intake, rhythm of dietary intake, eating behavior, and knowledge about diet.
- “Activity and media”: mount and style of physical activity, reasons/aims, and barriers for/to physical activity and support in physical activity. In the field of media consumption, parental mediation, attitudes towards advertising, parent-child conflict and the duration of media consumption are measured and analyzed.
- “Family life”: psychological variables of interest are disruptive behaviors in children and adolescents, family climate, shared activities, sleep, eating disorder symptoms, body dissatisfaction, stress, and family coherence.

**Table 2:** Overview of the questionnaire instruments according to the thematic modules of the STARKIDS intervention

Questionnaire	No. of items	Subscales	Reliability	Validity	Completer (about)
*Overall*					
Quality of Life Questionnaire for children (KINDL^R^), with obesity module *Original title: Fragebogen zur Erfassung der gesundheitsbezogenen Lebensqualität bei Kindern und Jugendlichen (KINDL*^*R*^*)* [23, 25–27]	24 plus 16 (obesity module)	Physical well-being, psychological well-being, self-esteem, family, friends, functioning in school/kindergarten	Cronbach’s *a* = .85	Convergent validity: parent report: *r* = .44–.63; child report: *r* = .33–.59 correlation with SDQ	Parent (child) Adolescent (self)
University of Rhode Island Change Assessment (URICA-S) [28]	16	Precontemplation, contemplation, action, maintenance	Cronbach’s *a* = .61–.84	n/a	Adolescent (self)
Parent Perspective University of Rhode Island Change Assessment -Short (PURICA-S) [24]	16	Precontemplation, contemplation, action, maintenance	Cronbach’s *a* = .30–.84	Criteria validity good to very good (exception subscale precontemplation)	Parent (self)
*Eating and drinking module*					
Dutch Eating Behavior Questionnaire – Children (DEBQ-C) [29]	20	Restraint eating, emotional eating, external eating	Cronbach’s *a* = .74–.81	n/a	Parent (child) Adolescent (self)
Rhythm of dietary intake	2		n/a	n/a	Parent (child) Adolescent (self)
Knowledge about nutrition	10				Parent (self) Adolescent (self)
Food Frequency Questionnaire (FFQ) – KiGGS version [30, 31]	114		n/a	*r* = .22–.69 correlation with DISHES nutrition interview	Parent (child) Adolescent (self)
*Activity and media module*					
MoMo-Physical-Activity-Questionnaire *Original title: Aktivitätsfragebogen des Motorik-Moduls* (MoMo-AFB, selected items) [32]	20		Intraclass correlation (ICC): *r* = .68	*r* = .29 correlation with accelerometer data	Parent (child) Adolescent (self)
Physical Activity, Exercise, and Sport Questionnaire *Original title: Bewegungs- und Sportaktivität Fragebogen* (BSA-F) [33]	10	Sports activity and physical activity	n/a	*r* = .32 with VO_2_max	Parent (self)
Physical Activity Enjoyment Scale (PACES) [34]	16		Cronbach’s *a* = .89–.93	*r* = .42 correlation with physical activity diary	Parent (child) Adolescent (self)
Self-concordance of sport- and exercise-related goals scale *Original title: Sport- und Bewegungsbezogene Selbstkonkordanz Skala* (SSK) [35]	12	Intrinsic, identified, introjected and extrinsic modes of motivation	Cronbach’s *a* = .70–.82	*r* = .25–.38 correlation with physical activity	Parent (child) Adolescent (self)
Basic Psychological Needs Questionnaire *Original title: Fragebogen zu Psychologischen Grundbedürfnissen*	9	Autonomy, relatedness, competence	n/a	n/a	Parent (child) Adolescent (self)
Exercise-related Support - Family *Original title: Sportbezogene Unterstützung aus der Familie (SU-F)* [36]	6		Cronbach’s *a* = .85	n/a	Parent (child) Adolescent (self)
Exercise-related Support - Friends *Original title: Sportbezogene Unterstützung von Freunden und Bekannten (SU-B)* [36]	5		Cronbach’s *a* = .89	n/a	Parent (child) Adolescent (self)
Barriers to physical activity	4		n/a	n/a	Parent (child) Adolescent (self)
Bernese Motive and Goal Inventory *Original title: Berner Motiv- und Zielinventar* (BMZI) [37]	26	Contact, competition/performance, distraction/catharsis, body/appearance, health, fitness, esthetics, and risk/challenge	Test-retest: .62–.83	Discriminant validity for acceptable	Parent (child) Adolescent (self)
Parental mediation [38–42]	51	Active parental mediation, restrictive parental mediation, social co-viewing/co-use, supervision, concept-oriented family consumer communication, socio-oriented family consumer communication	Cronbach’s *a* = .75–.94	n/a	Parent (self)
Attitudes towards advertising [38, 43]	7		Cronbach’s *a* = .80	n/a	Parent (self)
Parent-Child Conflict [42, 44]	3		Cronbach’s *a* = .69	n/a	Parent (self)
Duration of media consumption	2		n/a	n/a	Parent (child) Adolescent (self)
*Family life module*					
Strength and Difficulties Questionnaire (SDQ) [45, 46]	25	Emotional problems, striking behaviors, hyperactivity, troubles with peers, pro-social behaviors	Cronbach’s *a* = .55–.77 (self-evaluation)	n/a	Parent (child) Adolescent (self)
Family climate (items of the iFamily study) [47–49]	8		Cronbach’s *a* = .61–.83	n/a	Parent (self) Adolescent (self)
Shared activities (items of the iFamily study) [47, 49]	6		n/a	n/a	Parent (self) Adolescent (self)
Sleep (items of the KiGGS study) [50]	4		n/a	n/a	Parent (child) Adolescent (self)
Eating Disorder Examination Questionnaire 8 (EDEQ-8) [51]	8		Cronbach’s *a* =.93	*r* = .75 correlation with EAT-13	Parent (self)
Child version of the Eating Disorder Examination Questionnaire 8 (ChEDEQ-8) [52]	8		Cronbach’s *a* = .89	*r* = -?.65 correlation with body esteem scale	Adolescent (self)
Eating Disorder Inventory (EDI-2), subscale body dissatisfaction [53–55]	9	Only one scale used (body dissatisfaction)	Cronbach’s *a* = .84–.89 *r*_*tt*_ = .89/.94 (subscale)	*r* = .35 correlation with Beck Depression Inventory (subscale)	Parent (self)
Eating behaviors inventory – child, subscale dissatisfaction with body *Original title: Inventar zum Essverhalten und Gewichtsproblemen – Kind (IEG-K)* [56]		Only one scale used (body dissatisfaction)	Cronbach’s *a* = .88 (subscale)	n/a	Adolescent (self)
Weight Bias Internalization Scale (WBIS) [57, 58]	11		Cronbach’s *a* = .87 *r*_*tt*_ = .88 (8 weeks)	*r* = .2 for BMI-SDS *r* = -?.7 for self-esteem *r* = -?.77 for HRQoL (weight), acceptable construct validity in CFA	Adolescent (self)
Modified Weight Self Stigma Questionnaire (WSSQ) [59]	13	Modification of original scale to assess stigma by association	Cronbach’s *a* = .87 for original scale	*r* = -?.47 for HRQoL (weight)	Parent (self/child)
Body Image Avatars [60, 61]	3		These ratings have never been used before, for similar 2D avatars, a sample with female adult participants found *r*_*tt*_ = .88–.90 (5 weeks)	Rating corresponds with actual BMI: *r* = .89	Parent (child) Adolescent (self)
Parenting Self-efficacy questionnaire *Original title: Fragebogen zur Selbstwirksamkeit in der Erziehung* (FSW) [62]	9		Cronbach’s *a* =.78/.79	*r* = .63/.64 with parent behavior in risk situations	Parent (self)
Self-efficacy scale *Original title: Skala zur Allgemeinen Selbstwirksamkeitserwartung* (SWE) [63, 64]	10		Cronbach’s *a* =.78–.79	n/a	Adolescent (self)
Perceived Stress Questionnaire-20 (PSQ-20) [65]	20	Worries, tension, joy, demands	Cronbach’s *a* =.80–.86	*r* = -?.58 correlation with WHOQOL-Bref (global)	Parent (self)
Systemic Clinical Outcome and Routine Evaluation-15 (SCORE-15) [66]	15		Cronbach’s *a* = .89	Convergent validity shown by correlation with various measures	Parent (self) Adolescent (self)
*Socioeconomic outcomes*					
Mannheimer Modul RV (MRV), adaptation for STARKIDS	31		n/a	n/a	Parent (child) Adolescent (self)

*BMI-SDS* BMI standard deviation score, *CFA* confirmatory factor analysis, *EAT-13* Eating Attitudes Test – short version, *HRQoL* health-related quality of life, *n/a* not available, *WHOQOL-Bref* World Health Organization Quality of Life questionnaire, *VO*_*2*_*max* maximal oxygen consumption

#### Other outcomes

In addition to the aforementioned outcomes, demographic and physiological variables such as somatic diseases related to overweight and obesity and blood levels as well as motivational stages according to Junne et al. [24] are assessed. In addition, data of serious games and reflection tools provided by the e-health platform will be analyzed to complement the questionnaire data. For the health economics perspective, outcomes are costs and preference-based measures to elicit quality-adjusted life years (QALYs) as well as routine data from the statutory health insurance company AOK Baden-Wuerttemberg of invoiced health services.

### Participant timeline {13}

**Fig. 1:**
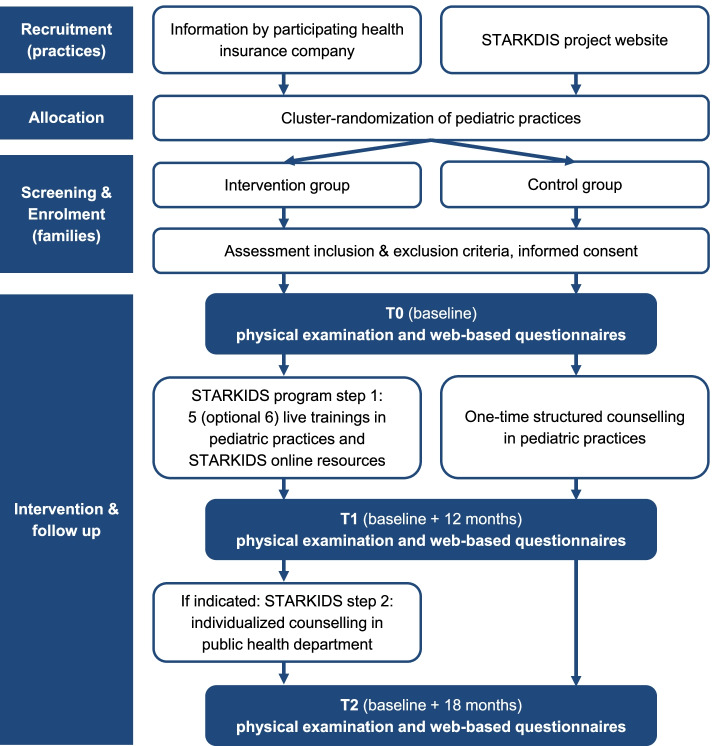
Participant timeline and schedule of enrollment, interventions, and assessments

The participant timeline is presented in Fig. 1.

### Sample size {14}

A total sample size of 1000 children and adolescents from 100 pediatric practices (on average 10 in each pediatric practice, with high variability of cluster sizes) was determined. This is based on a power calculation that takes dropout (adherence rate of 75%) and a cluster effect of 2 into account. With the projected final sample (750 individuals in 100 clusters), differences in proportions of participants that reach the target criterion (i.e., − 0.2 BMI-SDS_LMS_ reduction) between 14% (27% vs. 13%) and 17% (58.5 vs. 41.5%) are detectable with a maximum type I error rate of 5% and minimal power of 80% in a *χ*^2^ test.

### Recruitment {15}

In order to reach the target sample size, the recruitment of the current study is organized in collaboration with the participating health insurance company as well as the association of pediatricians of the state of Baden-Wuerttemberg. First, pediatric practices are recruited. All eligible pediatric practices are informed about the STARKIDS program and the accompanying study by the participating health insurance company. The project is also presented at different meetings of pediatricians by the study team. Additionally, a STARKIDS project website is launched with information about the study for potential families and pediatricians. Second, participating pediatric practices recruit families with children and/or adolescents with overweight/obesity: they go through their patient database to identify eligible children/adolescents and invite all eligible families to participate. Additionally, information material about the study provided by the study team is displayed in the practice (e.g., waiting room) to encourage eligible families to participate.

### Assignment of interventions: allocation

#### Sequence generation {16a}

The allocation sequence is randomly computer-generated.

Factors for stratification of participating pediatric practices are:

- Practice in an urban or rural area (according to postal code)
- Practice is a potential high recruiter or not (more than one participating pediatrician per practice or not)

Since practices are cluster-randomized, the participating families themselves are not allocated randomly but allocated through their pediatric practice.

#### Concealment mechanism {16b}

Not applicable, since pediatric practices need to know their allocation in order to receive the correct training for the study.

#### Implementation {16c}

The study team will enroll participating pediatric practices. The allocation of the pediatric practices is done by the evaluating site. Participating practices will recruit and enroll patients. Patients are automatically assigned to interventions depending on their respective pediatric practice being in the intervention or the control group.

### Assignment of interventions: blinding

#### Who will be blinded {17a}

Due to the nature of the study, testing the STARKIDS intervention versus a control intervention, participating practices cannot be blinded to allocation. During recruitment of families, practices are asked not to reveal their allocation to potential participating families until families have decided on their participation.

#### Procedure for unblinding if needed {17b}

Not applicable since there is no blinding.

### Data collection and management

#### Plans for assessment and collection of outcomes {18a}

Outcomes are assessed at the three measurement time points and collected web-based through the STARKIDS e-health online platform which prevents duplicate measurements. Table 2 shows the applied questionnaires according to intervention modules. Further measurements (e.g., height, weight, somatic comorbidities, and blood levels) are assessed in the pediatric practices at the three measurement time points. The primary outcome BMI-SDS_LMS_ is calculated using the children’s height, weight (both measured in the pediatric practices), age, and gender.

#### Plans to promote participant retention and complete follow-up {18b}

Participant retention and complete follow-up are promoted by the monitoring of the pediatric practices in which families are. This is supported by mechanisms of the e-health online platform remembering families of, e.g., the follow-up questionnaires.

#### Data management {19}

All data is assessed pseudonymized. Data will be recorded directly in the online portal for the study by either pediatricians and medical assistants (e.g., blood levels, examination results) or participating parents or adolescents (questionnaire data). Data entry forms can only be filled in once on the respective measurement point to avoid double data entries and cannot be changed once completed. Pseudonymized data is securely stored within the server infrastructure of the coordinating center. More information about data management procedures and security can be retrieved from the project’s data security and IT security policy. Data monitoring will be conducted according to the Center of Clinical Trials (ZKS Tuebingen) internal standard operating procedures (SOPs) and a dedicated monitoring manual for the study.

#### Confidentiality {27}

All personal information on enrolled families is only collected on paper versions and kept in locked units at the participating practice and later at the coordinating center to be archived. All information collected on the e-health online platform is under the families’ aliases and therefore does not contain any personalized information that enables the identification of the families. Data at the coordinating center is kept separate from the lists containing personal information of families and can only be retrieved by dedicated study team members or be inspected by study monitors for quality checking and verification.

#### Plans for collection, laboratory evaluation, and storage of biological specimens for genetic or molecular analysis in this trial/future use {33}

As common in medical practices, blood samples are taken from participating children and adolescents. Blood levels of interest for the study team are transferred into the online platform by medical assistants. Therefore, there is no need for the storage of biological specimens by the study team.

## Statistical methods

### Statistical methods for primary and secondary outcomes {20a}

For the analysis of both primary and secondary outcomes a frequency comparison *χ*^2^c tests accounting for dependencies among observations within clusters [67] as well as generalized estimation equations (GEE) approaches will be used. The latter regression models will incorporate adjustment for relevant baseline covariates as well as concurrent covariates. A dichotomous variable capturing membership in the control vs. intervention group will be included in these models to assess the treatment effect as well as—with interaction terms—differential effectiveness in subgroups.

Analyses are conducted using all observations with available data (intention-to-treat) as well as the per-protocol subset of observations with adequate consideration of potential differences in results between these two strategies. Maximal utilization of data from families that eventually drop out of the intervention program will be secured and adequate steps will be undertaken to obtain at least measurements of the primary outcomes from these families after participation discontinuation.

In addition, cost-effectiveness and cost-utility analyses are performed. Adjusted total costs per capita, QALYs, and differences in total costs and QALYs between the control and intervention group are calculated using mixed-effects linear regression models with robust standard errors adjusted for relevant covariates at baseline. To account for uncertainty with regard to costs and effects, nonparametric bootstrappings are performed. The cost-effectiveness acceptability curves of the intervention group are calculated based on the net benefit approach. Therefore, multilevel mixed-effects linear regressions are used based on the net monetary benefit for different willingness-to-pay (WTP) thresholds. A long-term perspective on the macrosocial added value of STARKIDS will be performed with the help of decision-analytic modeling or state-transition modeling.

### Interim analyses {21b}

Interim analyses are performed after the completion of the second measurement time point to evaluate step 1 of the STARKIDS intervention.

### Methods for additional analyses (e.g., subgroup analyses) {20b}

#### Qualitative interviews and focus groups

As an evaluation of the STARKIDS material, we conducted two focus groups and 9 interviews. The focus consisted of three parents of children with obesity and the children themselves. Materials from the STARKIDS program were shown and feedback was taken. The interviews were held at a later time point with five experts from a medical institution for children with obesity and four families being treated there. Materials (e.g., an educative movie, a serious game) were shown and feedback was taken.

#### Process evaluation

Participating pediatricians and medical assistants, as well as participating families are given questionnaires (tablet-based) to evaluate the STARKIDS program. The same items are given before and after the STARKIDS study as well as additional items about each participating family to assess their respective program fit. Items are rated on 5-point Likert scales.

### Methods in analysis to handle protocol non-adherence and any statistical methods to handle missing data {20c}

Protocol non-adherence will be accounted for through the use of covariates capturing pertinent aspects of such non-adherence in the regression models mentioned under the “Statistical methods for primary and secondary outcomes {20a}” section as well as separate analyses for observation groups with difference adherence levels. Potentially different results between these groups as well as a potential role of adherence as a predictor of treatment effectiveness will be transparently considered and accounted for.

Provided that missingness of data can be plausibly considered as missing at random, multiple imputation techniques will be used in order to avoid untoward loss of non-missing information that could occur through the use of other strategies (e.g., inadequate listwise deletion).

### Plans to give access to the full protocol, participant-level data and statistical code {31c}

The datasets analyzed during the current study and statistical code are available from the corresponding author on reasonable request, as is the full protocol.

### Oversight and monitoring

#### Composition of the coordinating center and trial steering committee {5d}

Coordinating center and steering committee:

- Enrolls pediatric practices in the study
- Supports pediatric practices in recruiting families (if needed) and providing information on the study
- Administrates lists with study center aliases and demographic information of participating practices
- Generates lists for pediatric practices for the allocation of participating families to study aliases
- Receives questionnaire results of participating families in the admin area of the e-health online platform
- Provides the insurance company AOK Baden-Wuerttemberg with the identifiers of participating practices to enable payment for the face-to-face trainings
- Provides the insurance company AOK Baden-Wuerttemberg with insurance membership numbers of the participating families for the pre-defined medical data from the families’ health insurance records.

#### Composition of the data monitoring committee, its role, and reporting structure {21a}

A data monitoring committee with a semiannual reporting structure via videoconference is implemented. The data monitoring committee is independent from the sponsor and competing interests.

#### Adverse event reporting and harms {22}

Participating practices and participating families can both report the occurrence of (severe) adverse events to the coordinating center through a study hotline or study email address. The study team manages reported (severe) adverse events.

#### Frequency and plans for auditing trial conduct {23}

Data monitoring will be conducted according to the Center of Clinical Trials (ZKS Tuebingen) internal Standard Operating Procedures (SOPs) and a dedicated monitoring manual for the study. The monitors will review the source documents as needed, to determine whether the data reported in the e-health online platform are complete and accurate. Forty planned monitoring visits are split between high recruiting pediatric practices and randomly selected pediatric practices. The trial steering committee meets at least monthly during the trial period and the data monitoring committee meets semiannual to review conduct throughout the trial period.

#### Plans for communicating important protocol amendments to relevant parties (e.g., trial participants, ethical committees) {25}

All important protocol amendments will be communicated to relevant parties (e.g., participating pediatric practices, ethics committee) through the coordinating center via newsletters or direct contact.

#### Dissemination plans {31a}

Trial results will be communicated by the funder, the participating healthcare insurance company AOK Baden-Wuerttemberg as well as the study team to the public and healthcare professionals through websites, newsletter, and publications.

## Discussion

The stepwise, combined face-to-face, and e-health-supported STARKIDS program is a low-threshold intervention program for families with children/adolescents with overweight or obesity. The program has several advantages: (1) the stepped procedure and the accompanying e-health aspects enable adaptions of the program to the individual families (e.g., concerning frequency and duration as well as focal points with regards to content), (2) the program is designed for the whole family and takes parents perspectives as well as adolescents perspectives into account, (3) face-to-face trainings in families local pediatric practices with their known healthcare providers facilitates access to the program and promotes adherence.

Potential issues in performing the study might arise from placing the face-to-face trainings in the pediatric practices, especially in light of the high burdens of pediatricians in the ongoing COVID-19 pandemic. They are however parents preferred professionals for broaching the topic of their children’s/adolescents’ weight and giving guidance [68]. Furthermore, face-to-face trainings are conducted by medical assistants in the pediatric practices, strengthening and expanding their roles in respective practices. Additionally, through the combination of face-to-face trainings and e-health components of the STARKIDS intervention, the program provides a timely presentation of prevention and intervention materials. Evidence for adolescents also suggests that especially health education, goal setting, and self-monitoring can be effectively delivered via websites [69]. In light of the ongoing COVID-19 pandemic in which obesity in children and adolescents has become an even more urgent health problem [70, 71], we therefore hope to support children/adolescents with overweight/obesity and their families in a healthy weight development.

### Trial status

The current protocol version is version 1 from November 2021. Recruitment and enrolment of participating families started on April 1, 2022. Recruitment will be completed when the planned sample size was achieved or after a 6-months recruitment period (can be extended due to obstacles through the COVID-19 pandemic).

## Data Availability

Any data required to support the protocol can be supplied on request.

## Abbreviations

BMI: Body mass index
BMI-SDS_LMS_: Body mass index – standard deviation scores (LMS method)
GCP: Good Clinical Practice
QUALYs: Quality-adjusted life years
TAU: Treatment-as-usual
WHO: World Health Organization
